# Editorial: Marine Microbes for Contaminant Bioremediation

**DOI:** 10.3389/fmicb.2021.762968

**Published:** 2021-11-02

**Authors:** Xuwang Zhang, Surajit Das, Ang Li, Qiao Ma, Liang Tan

**Affiliations:** ^1^School of Ocean Science and Technology, Dalian University of Technology, Panjin, China; ^2^Laboratory of Environmental Microbiology and Ecology (LEnME), Department of Life Science, National Institute of Technology Rourkela, Rourkela, India; ^3^State Key Laboratory of Urban Water Resource and Environment, Harbin Institute of Technology, Harbin, China; ^4^College of Environmental Science and Engineering, Dalian Maritime University, Dalian, China; ^5^School of Life Science, Liaoning Normal University, Dalian, China

**Keywords:** marine microbes, bioremediation, contamination, microbial community, oil spill, antibiotic resistance gene

Marine microbes are ubiquitous in the ocean environment and play a crucial role in numerous biogeochemical processes. With recent advances in molecular ecological approaches, the structure, diversity, and functional potential of marine microbial communities from seawater and sediment have been unveiled at a global scale (Sunagawa et al., 2015; Hoshino et al., 2020). However, urbanization and consequent industrialization of the landscape during the past decades have resulted in worldwide contamination of the oceans by substances such as petroleum hydrocarbons (PHCs), polycyclic aromatic hydrocarbons (PAHs), antibiotics, heavy metals, and excessive nitrogen and phosphorus. Particularly in coastal areas, these pollutants may accumulate in seawater and sediments, eventually posing a threat to marine ecosystems and public health. Marine microbes are the ideal candidates for the bioremediation of ocean contaminants, due to their diverse catalytic activities and ability to thrive under harsh conditions (e.g., hypersaline, low temperature, acidic or alkaline pH, and high pressure) (Dash et al., 2013; Beygmoradi and Homaei, 2017). Therefore, this Research Topic aims to contribute toward a better understanding of the roles and potential applications of marine microbes in contaminant bioremediation.

Marine oil spills and oil pollution are major global concerns and lead to serious adverse impacts on the economy and ecosystems. Bioremediation is considered as an efficient and environmental-friendly tool for the clean-up of oil-polluted areas (Mapelli et al., 2017; Varjani, 2017). To examine the potential of native microbial communities for bioremediation of oil spills, Bôto et al. collected multiple seawater samples from 47 sites along the NW coast of Iberian Peninsula, and exposed them to crude oil for 2 weeks. The results demonstrated that the oil-enriched microbial communities were dominated by hydrocarbon-degrading bacteria with no significant differences in geographical locations, such as *Alcanivorax, Pseudomonas, Acinetobacter, Rhodococcus, Flavobacterium, Oleibacter, Marinobacter*, and *Thalassospira*, which could represent prototype consortia for mitigating oil spills. Biosorption is an efficient way widely used in field remediation for the removal of pollutants from soil and water, and a vast array of biosorbent materials have been developed, including natural biosorbents (e.g., clays, chitosan), microbial biomass (e.g., bacteria, fungi, algae), and agriculture and industrial wastes (Vijayaraghavan and Yun, 2008; Yaashikaa et al., 2021). Martins et al. proposed a novel approach to bioremediation of oil spills by combining biosorbent with hydrocarbonoclastic bacteria. They found that corksorb, a cork-based biosorbent, could promote the growth of hydrocarbonoclastic bacteria and enhance the metabolic activity for alkane degradation. The native alkane-degrading bacteria in corksorb could also participate in hydrocarbons degradation, which may assist *in situ* bioremediation process. On the other hand, sulfate-reducing microorganisms may cause oil souring in oil reservoirs through production of H_2_S, while nitrate-reducing microorganisms can inhibit sulfate reduction that leads to biosouring mitigation. Understanding the structure and function of microbial community across different phases of biosouring and mitigation can improve mitigation measures. Dutta et al. suggested that mitigation strategies for biosouring could be improved by monitoring volatile fatty acid concentration and microbial diversity in oil reservoirs.

Antibiotics currently have caught global attention. Antibiotic residues discharged by human activities into the environment may alter the composition of microbial communities, and affect microbial ecological functions (e.g., nitrification, denitrification, and carbon metabolism) (Ding and He, 2010; Cycoń et al., 2019). The excessive usage of antibiotics can also lead to the enrichment of antibiotic-resistant bacteria (ARB) and antibiotic resistance genes (ARGs), which may render antibiotics useless and pose risks to ecological and human health (Zheng et al., 2021). Antibiotics and ARGs are frequently detected in estuarine and coastal environments across the world (Hatosy and Martiny, 2015; Zheng et al., 2021). Su et al. evaluated the antibiotic resistance status and their potential risk in coastal environment. The authors predicted the intrinsic ARGs (piARGs) in the coastal sediments collected from the East China Sea, and revealed the connections between piARGs and microbial community. They further suggested that the abundance-weighted average ribosomal RNA operon (*rrn*) copy number of microbial communities could be regarded as an indicator to assess the antibiotic resistance status.

This Research Topic will advance our knowledge regarding the field of marine microbes for contaminant bioremediation. In the current Research Topic, the impacts of oil pollution and ARGs on marine ecosystems were carefully investigated, and novel oil pollution bioremediation strategies by combining biosorbent with hydrocarbonoclastic bacteria were developed. On the other hand, the effects of marine microbes on oil biosouring and potential mitigation solution were also highlighted. Marine microbes have enormous capability for contaminant bioremediation, but there is still much to be explored. To increase the bioremediation potential of marine microbes, establishing adequate strategies to manage environmental conditions, illuminate genetic elements and optimize metabolic pathways will be helpful. Genetic engineering and manipulation in marine microbes may enhance bioremediation efficiency even further. Moreover, as the majority of marine microbes are unculturable under laboratory conditions, their functions and phenotypes remain largely unexplored, though some of them may play a critical role in degrading environmental pollutants (Bodor et al., 2020; Mu et al., 2021). Thus, developing efficient isolating and culturing strategies for the uncultured marine microbes may lead to the discovery of novel microbial resources for marine contamination treatment.

## Author Contributions

XZ wrote the draft of the editorial. All authors contributed to the reviewing, editing, and approval of the editorial.

## Conflict of Interest

The authors declare that the research was conducted in the absence of any commercial or financial relationships that could be construed as a potential conflict of interest.

## Publisher's Note

All claims expressed in this article are solely those of the authors and do not necessarily represent those of their affiliated organizations, or those of the publisher, the editors and the reviewers. Any product that may be evaluated in this article, or claim that may be made by its manufacturer, is not guaranteed or endorsed by the publisher.
